# Containing pandemics through targeted testing of households

**DOI:** 10.1186/s12879-021-06256-8

**Published:** 2021-06-09

**Authors:** André Voigt, Nikolay Martyushenko, Emil Karlsen, Martina Hall, Kristen Nyhamar, Stig William Omholt, Eivind Almaas

**Affiliations:** 1 Department of Biotechnology and Food Science, NTNU - Norwegian University of Science and Technology, Trondheim, Norway; 2 Department of Circulation and Medical Imaging, NTNU - Norwegian University of Science and Technology, Trondheim, Norway; 3 K.G. Jebsen Center for Genetic Epidemiology, NTNU - Norwegian University of Science and Technology, Trondheim, Norway

**Keywords:** COVID-19, Epidemic, Network spread, Computational model

## Abstract

**Background:**

While invasive social distancing measures have proven efficient to control the spread of pandemics failing wide-scale deployment of vaccines, they carry vast societal costs. The development of a diagnostic methodology for identifying COVID-19 infection through simple testing was a reality only a few weeks after the novel virus was officially announced. Thus, we were interested in exploring the ability of regular testing of non-symptomatic people to reduce cases and thereby offer a non-pharmaceutical tool for controlling the spread of a pandemic.

**Methods:**

We developed a data-driven individual-based epidemiological network model in order to investigate epidemic countermeasures. This models is based on high-resolution demographic data for each municipality in Norway, and each person in the model is subject to Susceptible-Exposed-Infectious-Recovered (SEIR) dynamics. The model was calibrated against hospitalization data in Oslo, Norway, a city with a population of 700k which we have used as the simulations focus.

**Results:**

Finding that large households function as hubs for the propagation of COVID-19, we assess the intervention efficiency of targeted pooled household testing (TPHT) repeatedly. For an outbreak with reproductive number *R*=1.4, we find that weekly TPHT of the 25% largest households brings *R* below unity. For the case of *R*=1.2, our results suggest that TPHT with the largest 25% of households every three days in an urban area is as effective as a lockdown in curbing the outbreak. Our investigations of different disease parameters suggest that these results are markedly improved for disease variants that more easily infect young people, and when compliance with self-isolation rules is less than perfect among suspected symptomatic cases. These results are quite robust to changes in the testing frequency, city size, and the household-size distribution. Our results are robust even with only 50% of households willing to participate in TPHT, provided the total number of tests stay unchanged.

**Conclusions:**

Pooled and targeted household testing appears to be a powerful non-pharmaceutical alternative to more invasive social-distancing and lock-down measures as a localized early response to contain epidemic outbreaks.

**Supplementary Information:**

The online version contains supplementary material available at (10.1186/s12879-021-06256-8).

## Background

Protocols for testing the presence of COVID-19 (Coronavirus disease of 2019) were available [1] well before it was declared a pandemic by the World Health Organization on March 11, 2020 [2]. In contrast, despite an unprecedented international effort to develop vaccines against SARS-Cov-2 (Sudden Acute Respiratory Syndrome Coronavirus 2), the large-scale distribution of a successful vaccine has still not fully materialized world-wide. Due to time-consuming clinical trials that any vaccine candidate has to pass, this asymmetry is likely to characterize the majority of new virus pandemics that may emerge in the future. Thus, exposure to a new virus pandemic is characterized by at least a 12 month window when accounting for the time lag between production launch and appearance of a substantial intervention effect from a vaccine [3]. During this time, we are forced to curb spread through exploiting interventions provided by population testing and other non-pharmaceutical measures, such as invasive social distancing, and community, city and region-wide lock-downs [4–9]. However, these interventions carry vast societal costs [10].

The ability of a given test regime to inhibit spread has a substantial impact on the need for other measures: the greater the effect of testing, the fewer social restrictions need to be invoked, some of which carry huge societal costs compared to the expenses attached to testing. Thus, the identification of optimal test regimes in terms of efficacy, logistical feasibility and economic cost as a function of infection dynamics, deserves close attention. Here we show that, for any pandemic-causing virus behaving similar to SARS-CoV-2 [11], localized targeted testing of large households and subsequent quarantining of positive cases, is a highly efficient strategy for getting the infection dynamics under control.

Most countries have implemented a COVID-19 test regime targeting symptomatic cases combined with contact tracing. In addition to the substantial economic costs related to contact tracing, there are several inherent problems associated with this approach. First, the time from exposure to onset of symptoms is estimated to be 2-12 days, with a mean of 5.5 [12]; Second, there is typically a multi-day lag after the first onset of symptoms until a test is performed; Third, voluntary opt-in symptomatic testing will leave many people with weaker symptoms untested for a multitude of socioeconomic reasons [13]; Finally, the obvious inability to systematically identify the considerable fraction of asymptomatic spreaders [14–16] implies that an important infection source is not targeted. Even with an exemplary implementation of symptomatic testing and contact tracing, a large number of the uncovered positive cases will have spent a significant portion of their infectious period unmitigated, and only a limited number of the potential disease-transmission chains in a society will be severed. This warrants the search for alternative feasible test regimes lacking the shortcomings of symptomatic testing.

One of the primary obstacles to widespread regular testing of large populations is limited laboratory capacity for analyzing samples. One possible strategy to mitigate this obstacle is based on *pooling* samples: rather than analyzing each sample individually, parts of samples from different individuals (forming a pool) are combined and analyzed together. The return of a positive result will be for the group as a whole without specifying which individual(s) in the pool are infected [17]. If appropriate, a positive pooled test can then be followed up by individual analysis of the remaining sample from each person in the pool in order to identify which of the pool participants are infected. An important concern when conducting pooled testing is the dilution of samples, leading to a possible loss in sensitivity and an increased number of false negatives. For PCR (polymerase chain reaction) testing for COVID-19, it has been found that pools of up to 64 individuals can be done with minimal loss of sensitivity [18].

## Methods

### Identification of demographic and epidemic correlates

**Fig. 1:**
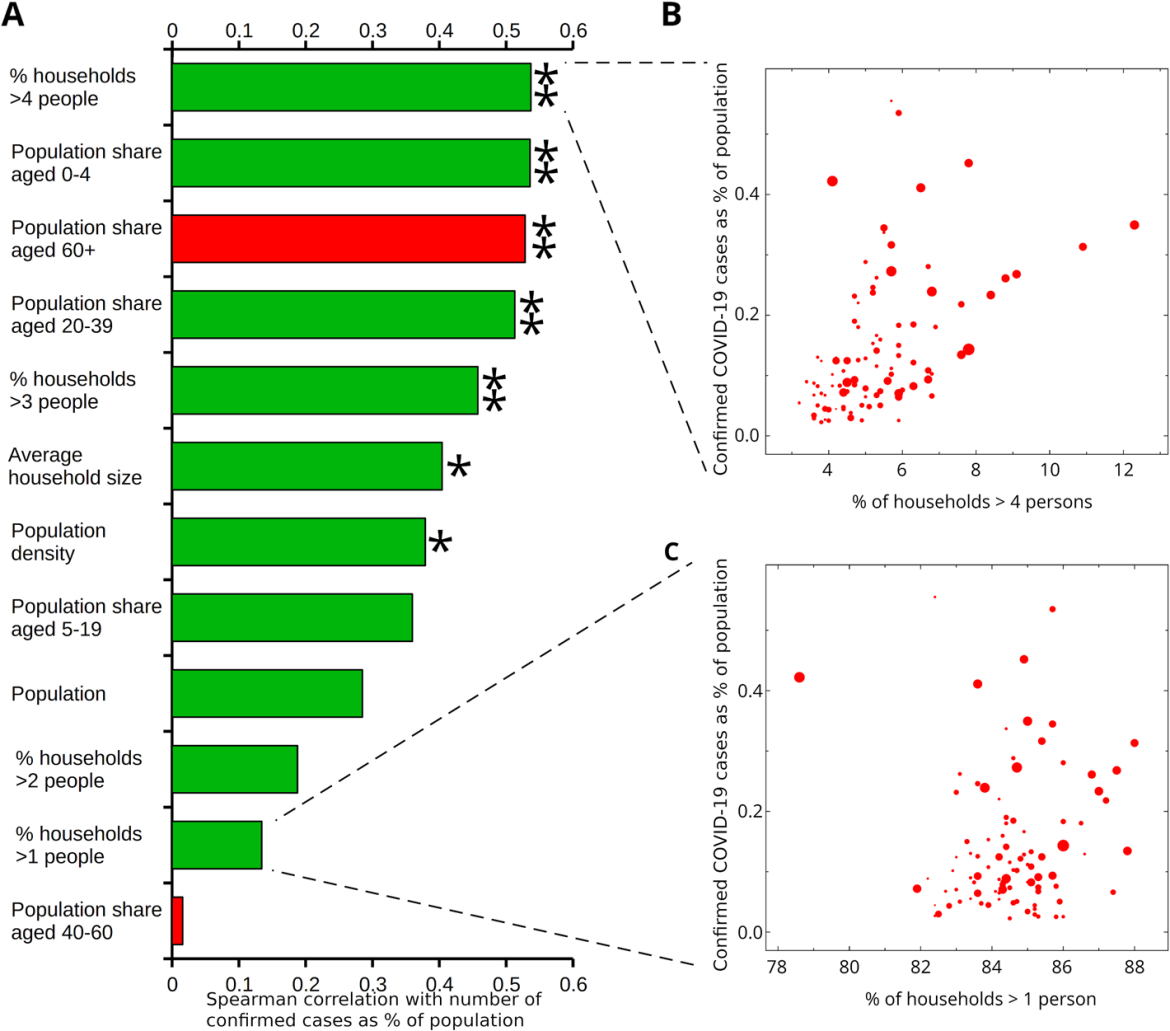
Simulation results and effectiveness of targeted pooled household testing (TPHT) on COVID-19. **(A)** Fitting the model to Oslo hospitalization data. We plot the mean predicted number of hospitalizations (black) and confidence interval (2*σ*, dashed). Actual Oslo hospitalizations (red) were used as calibration (until May 15th). Hospitalization data May 15th- August 30th (green) were not used to determine model parameters. **(B)** Effect of TPHT in response to a sudden rise in cases (reaching 1,000 symptomatic individuals), assuming general infectivity parameters similar to those of Oslo in late May 2020 but with 75% increased infectivity of random contacts giving *R*=1.2. (C) Predicted number of deaths and infections for different TPHT testing fractions corresponding to panel **(B)**, relative to no testing. Panels **(D)**-**(F)** use same parameters as panel **(B)**, except with a 113% increased infectivity of random contacts giving *R*=1.4. **(D)** Effect of test frequency and fraction on *R*, for TPHT (left) and random pooled household testing (right). Dashed and solid lines indicate isoclines for *R*=1 and constant test density, respectively. Optimal point is marked with red circle. **(E)** Response of weekly TPHT to varying city size. We scale the population of the baseline Oslo model (*γ*=1) to generate larger (*γ*>1) or smaller networks with household, school, daycare, workplace and nursing home size-distributions unchanged. **(F)** Response of weekly TPHT to changes in distribution of household size, relative to the baseline Oslo model (*α*=0). For *α*>0, a portion of households are each split into a new pair, yielding a smaller average household size than the baseline. For *α*<0, a portion of the households are pairwise merged, yielding a larger average household size than the baseline. Household, school, daycare, workplace and nursing home size-distributions are kept unchanged. (G) COVID-19 stopping time (number of days until symptomatic cases are reduced by 75%) in response to changing days between tests (left) and fraction of weekly TPHT tests (right). Stopping times longer than 100 days are truncated

In order to get a general idea of population-wide demographic parameters relevant to spread of COVID-19 that could serve as the basis for mitigation strategies, we pulled detailed demographic data from France’s National Institute of Statistics and Economic Studies (INSEE) [19] combined with epidemiological data from the French Agency for Public Health (Santé publique) [20] for the 96 European departments of France (Fig. 1). France was chosen as a target for this type of analysis for three main reasons: First, while both hospitalization and demographic data were available for many developed countries, the French data are readily accessible in bulk machine-readable formats and in high level of detail; Second, both hospitalization and demographic data were aggregated for the same administrative subdivisions (departments), allowing exact matching; Third, France saw extensive spread of COVID-19 relatively early by European standards.

The INSEE data set provides information for geographic coordinates, total population, surface area, density, household size and age distribution for each department of France. In order to get a general idea of how each of these demographic measures relate to epidemic spread, we used the Spearman rank correlation coefficient with hospitalization numbers across all French departments located in Europe as a guiding metric.

### Building a municipality IBM network

We generate a high-fidelity individual-based model (IBM) for a single municipality by creating a set of individuals corresponding to the population *N*_*m*_ of that municipality. To each individual, we assign a list of personal attributes, some of which are immutable (such as age) while others (such as disease state) change with time.

These individuals are organized into a multi-layer network model, consisting of nine layers. Further, an individual can only be present in a single of the layers b)-h). The layers are: a) Household, b) Day-care, c) Primary school, d) Secondary school, e) High school, f) Workplace, g) Nursing home, h) Hospital, and i) Generic contact network. Layers a)-g) each consist of many groups, and each individual is only member of at most one group in each layer. A group is designed as a *k*-clique, i.e. where all members of a group are in contact. With the exception of nursing home residents, all individuals are present in layer a), and in *one* of layers b)-h). Layer i) is different from the rest: It consists of a single group of which all nodes are members. Instead of functioning as a clique where each node has an equal chance of coming into contact with each other node, each node is assigned a personal activity level which indicates the possible number of interactions it can enter into each day, in order to represent how highly social individuals can act as potential super-spreaders.

Statistics Norway (SSB) [21] collects detailed data on the composition of households (both by number of residents and family type), on the number and size of schools, and number of employers by amount of employees, as well as age pyramids for the whole population. Using the API (application programming interface) provided by SSB, we were able to obtain relevant demographic parameters for any arbitrary Norwegian municipality. Where municipal-level data is unavailable, such as number of staff at educational and care institutions, we default to national averages.

### SEIR-type epidemiological dynamics

Each individual in the IBM model is either healthy, in various states of infection, or recovered from the disease: Susceptible (S); Exposed (E); Infected, asymptomatic (Ia); Infected pre-symptomatic (Ip); Infected, symptomatic (Is); Hospitalized (H); Intensive care (ICU); Recovered (R); or Dead (D). The different states and their possible transitions are captured by the state-transition schematic of Supp. Figure 2.

The default state is susceptible, in which an individual will remain indefinitely unless exposed by contact with an infected direct contact in any layer. For each infected individual in a susceptible individual’s daily contact network, there is a probability (depending on the type of layer, see Supp. Table 1) that the susceptible individual enters the exposed state. Once exposed, an individual’s progression through the states of infection is no longer dependent on network dynamics, but rather on state transition probabilities determined by that person’s age [22], as well as the distribution of each disease state duration (Supp. Table 1).

At each time point, an individual will store four points of information about their progression through the SEIR states: current state, date of last change of state, next state, and date of next change of state. Each day, the model updates the states as needed. Upon entering a new state, the model selects the following state according to probabilities and waiting times specified in Supp. Figure 2 and Supp. Table 1. Once the next state is determined, the duration for which the individual will remain in the recently entered state is generated according to a formula of one day plus a Poisson distributed variable determined by that state’s specific typical duration *λ*. The date of next change is set accordingly. Note that our SEIR-model uses age-stratified transition rates, where the age of an individual decides which of the age groups that individual is part of. Supp. Table 1 shows the rates used for the different SEIR transitions of Supp. Figure 2.

Each individual’s SEIR state determines to what extent they spread the disease in two different ways. The first is in determining the behavior of a given individual, with symptomatic individuals removed from non-household layers, and hospitalized individuals similarly removed from their households. In addition, the infectiousness of a given type of contact depends on whether an individual is symptomatic, pre-symptomatic (more infectious) or asymptomatic (less infectious).

### Model calibration

**Fig. 2:**
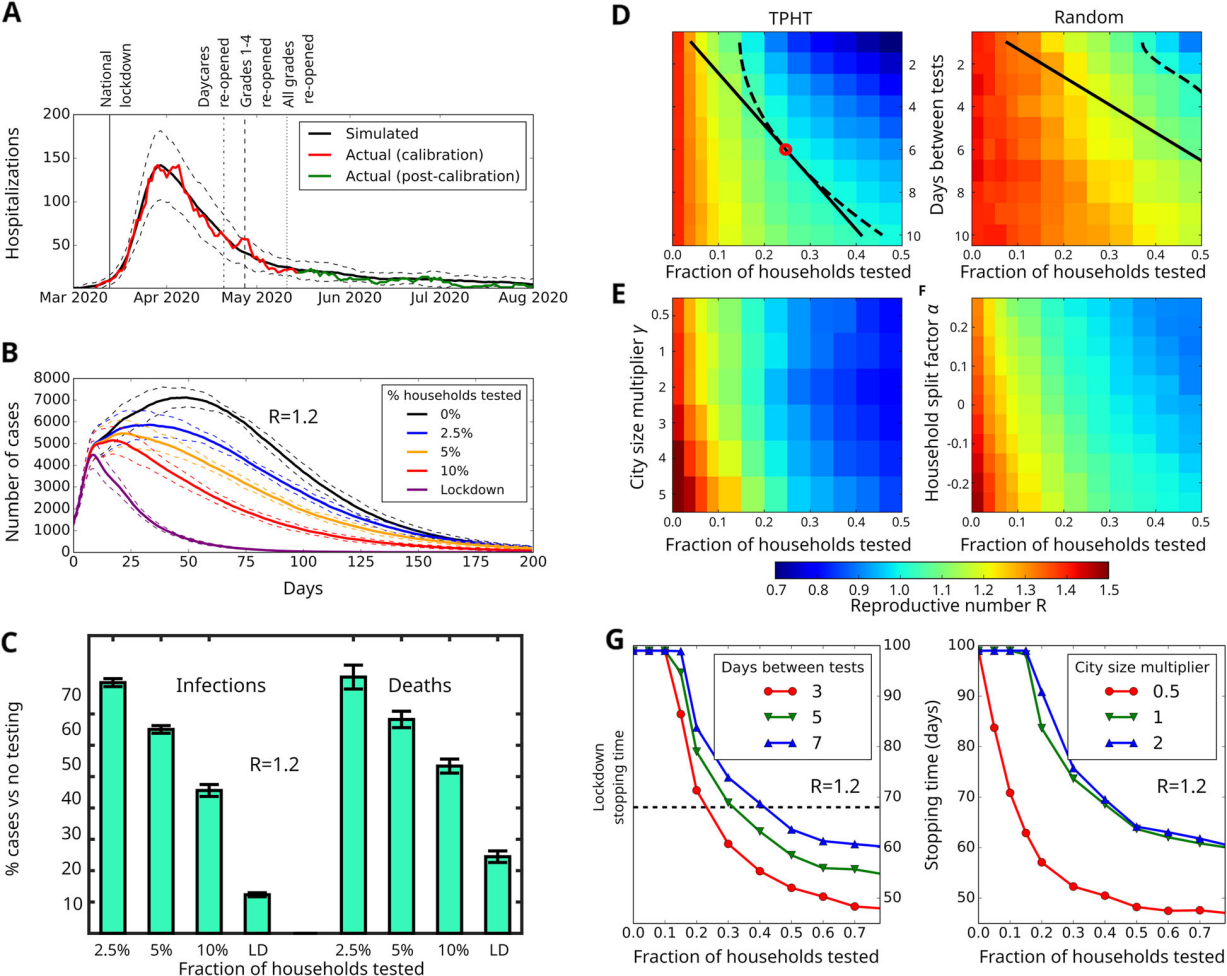
Demographic parameters associated with COVID-19 spread in France. **(A)** Correlation histogram (positive, green; negative, red) shows larger household sizes significantly correlated with levels of COVID-19 hospitalizations. Single (double) star indicates Bonferroni-corrected significance *P*<0.01 (*P*<0.001). Panels **(B)** and **(C)** show scatter plots of confirmed cases (as percentage of population) for the 96 departments of European France as function of the percentage of households larger than four persons and one, respectively. Size of markers is proportional to the population of each department

While transition probabilities and state durations could be set on the basis of clinical data, no direct data for the probability of infection by type of interaction was available. Therefore, infection probabilities for each layer needed to be determined indirectly by fitting epidemic curves to hospitalization data. While available, we rejected confirmed case counts as an accurate descriptor of epidemic spread during the initial months of the pandemic, as the gradual expansion from a limited testing capacity had as a consequence that the ratio of confirmed to actual cases would change with time. Using hospitalization data for Oslo [23] during the 2.5-month period from March 1st to May 15th, 2020 as a target (Fig. 2A, red line), we calibrated the infectivity of each layer by subjecting the model to the same course of shutdowns and re-openings as those mandated by Norwegian authorities in the same period. We implemented a manual-fitting procedure for the simulated hospitalization curve versus the actual data by only adjusting the infection probabilities until an acceptable fit was reached according to the following criteria: (1) slope-matching with both increasing and decreasing slope of the empirical data (Fig. 2A, red curve), (2) the location (date) and duration of the peak of the empirical hospitalization data, (3) actual hospitalization levels-fitting between confidence intervals for the duration of the calibration simulation. Additionally, we checked that (4) the fraction of computed household infections was within the 35-45% band seen in officially reported weekly values.

### Implementation of intervention measures

A high-fidelity IBM allows for the implementation of detailed interventions, where contact patterns of a selection, or all, of the individuals may be modified. It is thus straightforward to conduct computational experiments where some or all schools are closed or partially closed, various levels of social distancing are included, or testing and vaccination is administered. The most basic intervention strategy consists in locking down various IBM layers. How this is implemented in detail depends on the layer:

- For day care facilities as well as schools we specify an age-dependent cut-off, such that children above the specified age are temporarily removed from the layer. This allows us to simulate a partial shutdown of schools with a gradual re-opening as implemented by Norwegian authorities in the spring of 2020.
- In the work layer, we implement a partial shutdown by specifying that a fraction of cliques, intended to represent workplaces where work-from-home is feasible, are disabled.
- In the random contact layer, lockdown and general social distancing measures are implemented by reducing the daily number of contacts by a specified fraction.
- The household and nursing home layers are never disabled.

In addition to restrictions on the general population, the quarantining of an individual is represented by disabling workplace, school, and random layer spread for that individual. By default, symptomatic individuals will automatically go into quarantine when symptoms begin to manifest (i.e, when transitioning to the symptomatic state). When simulating testing protocols (for details see Supp. text), asymptomatic and pre-symptomatic individuals are similarly entered into quarantine upon testing positive, as are members of their households. In the absence of testing, asymptomatic and pre-symptomatic individuals remain infectious in all layers in which they are ordinarily present.

In this article, we propose and evaluate a testing and quarantine protocol that we call Targeted Pooled Household Testing (TPHT), defined by regular and scheduled pooled testing [24] of the largest households in a region followed by quarantining of the entire household upon a positive pooled test. We begin by both setting a test capacity (in number of households/pools) and an interval *i* between tests. The next step is to identify the largest households in the population and randomly assign a fixed date of first test between zero and *i* to each household. For example, setting *i*=7 would correspond to giving each household a specific day of the week on which it would then be subjected to a pooled test, which returns a positive result if any of the members in the household are in the Ia, Ip or Is states. From then on, all members of the household remain in quarantine until the household as a whole returns a negative test (assuming recovery entails immunity, confirmed recovered individuals could be exempt earlier, as whether or not an immune individual is quarantined has no impact on spread).

Households are a promising target for pooled testing for several reasons. First, we find a close association between the percentage of large households and prevalence in an area (see “Results” section). As a risk factor, household size also forms a clear criterion which is objectively quantifiable and difficult to address by other means. Due to the high probability of within-household transmission and the difficulty of avoiding contact in close familial relationships, in particular when involving children, robust disease control would likely entail whole-household quarantine even if only one member is initially identified as sick. The need for a household-wide quarantine reduces the need to perform follow-up analysis of individual samples if this becomes prohibitive due to high prevalence. Even very large households are well within the limits of reasonable pool sizes in order to avoid loss of sensitivity, with 16 samples in a pool still maintaining a sensitivity of 96% [18].

### Sensitivity to demographic parameters

To assess the generality of our results to other population regions, we investigated the effect of independently varying the population size (Fig. 2F) and the household size-distribution (Fig. 2G) while conducting weekly TPHT (for details on how we adjusted these characteristics, see Supp. Text). Using a population multiplier (*γ*), we scaled the size of our base-line model (*γ*=1) while keeping all other demographic distributions unchanged.

To investigate how changes to the household-size distribution impact the ability of TPHT to curb spread, we introduced a household-split factor *α* that determines if a pair of households should be joined (*α*<0) or a single household should be split into two (*α*>0), while keeping the population size unchanged (see Supp. Text for details).

## Results

### Epidemiological observations

In order to reduce the reproductive number *R* of a spreading process, network theory has shown that it is effective to immunize high-connectivity nodes (hubs) in heterogeneous systems [25, 26]. Thus, the detection and isolation of hubs in social interaction networks can be considered the cousin of vaccination: while it does not prevent the primary infection, it does prevent secondary infections. Anticipating that private households are major hubs for the spread of COVID-19, we analyzed in-depth contact-tracing data available for 5,531 of the laboratory confirmed cases occurring in Norway for a 30-week period after the outbreak. The data confirm the most likely location for exposure to be private households (37.4*%*) [27]. A recent report from the UK Scientific Advisory Group for Emergencies (SAGE) also emphasizes the clear connection between increasing size of a household and increasing risk of infection and mortality [28].

Calculating the correlation between available demographic variables and the extent of COVID-19 hospitalization in France, we find that the strongest correlates are dominated by household and household-related variables (Fig. 1A). In particular, the strongest correlated variable is the fraction of households with more than 4 persons (Fig. 1B), in contrast to households with more than 1 person (Fig. 1C). Similarly, the population shares of young adults (ages 20−39 years) and preschool children are amongst the four strongest positive correlates, indicating a link between families with children and COVID-19 spreading. In contrast, the population share of older people (ages 60+ years) is strongly negatively correlated with the spread of the disease.

### Simulation results

The model is able to obtain a close fit with the hospitalization data used for calibration (Fig. 2A, red line). Additionally, the parameters identified also prove predictive within confidence intervals for the period from mid-May to at least late August (Fig. 2A, green line),resulting in a predicted household infection fraction of 45%. In particular, the identified parameters (Supp. Table 1) correspond to an expected reproductive number of *R*≈0.9 for the period from May 15th until the end of June. Assured by the above validation results, we investigated the ability of weekly TPHT to contain an outbreak of 1,000 symptomatic cases in Oslo, a city with ∼700,000 inhabitants (see Supp. Text for details). We tested the infection spread situation with *R*=1.2 (Fig. 2B), obtained by increasing the chance of infection in the random contact layer by 75%. This is a little more than the estimated *R*=1.1 value for Oslo in September, 2020 [29]. We find that a TPHT involving a mere 2.5*%* of the households is able to noticeably reduce the number of cases (see Supp. text for details on TPHT selection). A weekly 10% TPHT-level reduces the number of infected by ∼55*%* and dead by 45% relative to no testing (Fig. 2C). It should be noted that the number of PCR-based tests associated with a 10% pooled TPHT-level is the same as what is necessary to individually test 5% of the Oslo population weekly.

### Impact of testing quantity

Assuming a situation with an *R*=1.4, similar to the experience of many European countries during fall 2020 [30], we explored the ability of TPHT to reduce *R* by varying the frequency and fraction of households to be tested (Fig. 2D). We determined the optimal balance between frequency of testing and number of tests needed to achieve the critical point *R*=1 by finding the density of daily TPHT that intersects the *R*=1 isocline to be *ρ*=*x*/*y*=0.03, with *x* being the fraction of TPHT performed per *y*-day interval. The model predicts that the optimal allocation to contain the epidemic with *R*=1.4 is to implement a testing regime where the 18% largest households are tested every six days (see Supp. Text, “Description of the TPHT process”, for details), corresponding to a number of tests equalling 3% of households every day. In comparison, we find that regular pooled testing of randomly selected households would require an infeasible number of tests to obtain an appreciable reduction in *R* (Fig. 2D), with a weekly schedule requiring well over 50% of households being tested each week. This illustrates that the prioritization of larger households in TPHT leads to a dramatic impact on *R*, allowing for an effective suppression of infection spikes with a moderately ambitious weekly test capacity (Fig. 2B).

When faced with an outbreak, its estimated duration is an important parameter for health authorities when planning mitigation. We define the outbreak stopping time as the duration until the outbreak is at a 25% level relative to when interventions were implemented. First we computed the stopping time associated with the Norwegian national lockdown regime in effect from March 13th (Fig. 2A) by extending the lockdown indefinitely without further re-openings. We find that this situation corresponds to a stopping time of 68±3 days.

For the outbreak in Fig. 2B, we measured the stopping time as function of TPHT fraction for different test frequencies (Fig. 2G, left panel). Reaching a testing fraction of 20−25*%* of the households dramatically reduces the stopping time as long as the testing frequency is at least once a week. However, more frequent tests significantly improves the calculated stopping time. Our analyses further suggest that for viral variants with increased contagion among children and youth, and if infected people do not isolate immediately upon manifestation of symptoms, the stopping time is only weakly dependent on the test frequency (see Supp. Figure 4)

### Sensitivity to demographic parameters

As shown in Fig. 2E, we find that larger cities need a larger TPHT fraction in order to bring *R* down to the same amount as in a smaller city, even though contact densities at the individual level remain unchanged. A likely explanation for this is the presence of scale-free spreading dynamics in our model, the impact of which has previously been shown to increase with larger network sizes [31]. The stopping time for weekly TPHT in three different population sizes (Fig. 2G, right) drops rapidly with increased TPHT test fraction until reaching a value of 0.3, for which it has achieved near saturation. For both measures (R and stopping time), we find that the main effect of TPHT remains consistent, with a stronger initial effect on spread (per test) at lower test fractions, but with diminishing returns as the proportion of households tested increases.

As anticipated, we find that *R* increases with the proportion of large households (Fig. 2F). By increasing the weekly test fraction, one may countermand this effect, and application of TPHT is still capable of reducing *R* to below unity, albeit requiring a substantially larger amount of testing (Fig. 2F). Thus, even though the demographic characteristics of a city has a noticeable impact on *R*, Fig. 2D-G indicate that TPHT remains capable of substantially reducing *R* for modest test fractions across a variety of city sizes and household compositions.

It is reasonable to assume that not every household would be willing to participate in the TPHT scheme. Investigating different scenarios with a varying degree of compliance (Supp. Figure 6), we find that the effect of TPHT remains fairly consistent for situations of less than perfect compliance. Even for participation rates as low as 50%, a testing capacity of 10% weekly (i.e. testing half of the 20% largest households) performs quite close to the ideal or near-ideal compliance scenarios (testing all of the 10% largest households), as there still remains a substantial number of households large enough to be at elevated risk that are still available for testing. However, as the test fraction increases, the *R*-curve begins to flatten, an effect which is more pronounced for lower compliance rates (as there are fewer large households available to enroll in testing). It follows that ensuring higher compliance with a TPHT program becomes more important in scenarios where a larger testing fraction is necessary.

## Discussion

In addition to factors such as testing frequency, household-size distribution and city size, the effectiveness of the TPHT strategy also depends on the properties of the infectious agent. One of the challenges with analyzing COVID-19 is related to the uncertainty in the details of its manifestation in infected people, such as incubation time, infectiousness in different age groups, number of days with infectiousness pre- and post-symptoms, to mention a few. While the results presented in Fig. 2 reflect the current best choice of COVID-19 parameters, we have analyzed the effect of TPHT using several estimates for these parameters at different times during the pandemic. A best-choice parameter set from August 2020 (Supp. Table 1, red) predicts that TPHT will have a much stronger effect (Supp. Figure 4) compared the results in Fig. 2.

Based on our analyses, we observe that in particular the number of days that a person is pre-symptomatic infectious will impact the ability of TPHT to reduce *R*: If this window is narrow, it is necessary with an increased frequency of TPHT to obtain a marked reduction in *R*. In the simulations, we have made the quite conservative choice that infected people will immediately self-isolate when they become symptomatic. As a consequence, the time window for TPHT to uncover pre-symptomatic infected people is relatively short. In reality, perfect compliance with self-isolation as soon as potential symptoms are experienced is quite unlikely since few of the COVID-19 symptoms are specific to the disease. If self-isolation is delayed by only a few days, the ability of TPHT to reduce *R* is markedly improved (Supp. Figure 4).

Additionally, we chose to implement reduced infectivity and infectiousness in children and young adults, as well as for asymptomatic infections. While these assumptions seem reasonable given current data, it is possible that viral strains could appear with increased infectivity and infectiousness for these groups. Since TPHT is a proactive testing strategy focused on larger households, in which the presence of children and young adults is quite common, TPHT would display improved effectiveness against such strains.

As a baseline, we have assumed a generally ideal implementation of TPHT with both 100% sensitivity and compliance. While pooled testing with pool sizes comparable to typical large households maintain a very good sensitivity (over 95%) for ordinary PCR protocols, there may be other obstacles to an ideal TPHT implementation. Traditional deep nose swabs commonly used for COVID-19 and other respiratory infectious disease are unpleasant and not necessarily appropriate for regular use or for use on small children. It might therefore be expedient to either only include household members above a certain age, or use less invasive but possibly less reliable tests. However, either of which would risk infection passing by undetected despite testing at the expense of loss of power of TPHT.

The fundamental principle behind TPHT is leveraging heterogeneity in the spreading potential of different segments of the population in order to focus resources on at-risk groups. Consequently, the marginal effect of adding testing capacity decreases as the test fraction increases and efforts move away from more extreme individuals to more average ones. The gap between TPHT and random testing (or other hypothetical testing strategies) necessarily narrows as the test fraction increases and the selected populations begin to overlap. If the critical test fraction (i.e. the fraction necessary to reach *R*=1) increases, either due to a more infectious baseline or imperfections in the implementation (such as poor compliance), the relative benefits of TPHT would be reduced accordingly.

It is therefore important to keep in mind that TPHT has limited potential as an isolated disease control measure, but is more suitable to implementation as part of a wider combination of measures. Due to the likely reduction in maximum potential benefit from a TPHT protocol in the event of imperfections in the implementation, ensuring that said implementation comes as close as possible to the ideal scenario is likely to be key to the success of a TPHT program. Putting in place sufficient logistics for the reliable collection and processing of test samples should be combined with information programs to allow the public to appreciate the benefits of participating in and complying with TPHT.

## Conclusions

In summary, we have found that the implementation of a TPHT regime is broadly effective in breaking infection chains before their spread potential has been realized. As a TPHT regime can be combined with a wide range of economically benign social distancing measures, our results suggest that it represents an attractive part of the toolkit for bringing a pandemic outbreak under control without inducing the societal costs of large-scale quarantines, lock-downs and curfews.

## Supplementary Information

**Additional file 1** Supplementary text. An additional text file that describes the software in detail, contains parameter tables, software availability, and additional figures.

## Data Availability

All data generated or analysed during this study are included in this published article and its supplementary information files. Contact Eivind Almaas, eivind.almaas@ntnu.no, for further data requests.

## Abbreviations

API: Application programming interface
COVID-19: Coronavirus disease of 2019
IBM: Individual-based model
PCR: Polymerase chain reaction
SAGE: UK Scientific Advisory Group for Emergencies
SARS-CoV-2: Severe acute respiratory syndrome coronavirus 2
SEIR: Susceptible-Exposed-Infected-Recovered
SSB: Statistics Norway
TPHT: Targeted Pooled Household Testing
