# Supplementary material for: Containing pandemics through targeted testing of households
Source: BMC Infect Dis. 2021 Jun 9;21:548. doi: 10.1186/s12879-021-06256-8 (PMC8189703; doi:10.1186/s12879-021-06256-8)
Supplement: Supplementary file 1 — Additional file 1 Supplementary text. An additional text file that describes the software in detail, contains parameter tables, software availability, and additional figures. [file 12879_2021_6256_MOESM1_ESM.pdf]

Supplementary Text

Description of individual-based modeling (IBM) software 2
Stochastic IBM network with SEIR epidemiological transmission dynamics 3
Structure of the municipality IBM network 3
Population size scaling 6
Variation of household-size distribution 6
SEIR-type epidemiological dynamics 8
COVID-19 testing of (sub-) population 9
Base-line fitting of IBM to empirical data 10
Description of the TPHT process 12
Selection of households 12
Pooled household testing and quarantine 13
Estimation of R for a choice of simulation parameters 13
Simulation of outbreaks 16
Software availability 17
Supplementary Figure 4. Effect of TPHT for alternative COVID-19 disease parameters 18
Supplementary Figure 5. Household-size histograms. 20
Supplementary Figure 6. Effect of compliance on the efficacy of TPHT. 22
Supplementary Table 1. Parameters used in simulations 23
Supplementary Table 2. Demographic data used to generate layered network. 26
Supplementary Table 3. List of 15 demographic variables studied in epidemic data from Santé publique 27

Description of individual-based modeling (IBM) software

When selecting and designing our computational modeling framework, we have explicitly taken into consideration the following aspects: (A) It should be possible to directly test a wide variety of interventions, such as partial workplace and school closings, social distancing, and testing with subsequent isolation of infected individuals. (B) Each infected person should have a disease-state development where the stages have realistic time delays. If a lag in response time is not included, we anticipate that all estimated response times to interventions will be markedly wrong. This is especially evident for the correct estimation of R, and thus, the capacity to determine how quickly R responds to interventions. The mentioned challenges are inherent to most metapopulation-based models, whereas an IBM approach is not hampered with such issues.

Guided by the above reasoning, we have developed a complex system modelling framework to describe the spread pattern of COVID-19 in mainland Norway. The model design and build features are chosen with the intent that it can be used to carefully assess a multitude of relevant intervention strategies. Briefly, the model is based on using an IBM based on complex network theory for each Norwegian municipality. The features of the model’s layers and each individual all adhere to high-resolution demographic data specific for the respective municipality. The disease-state of each individual follows an epidemiological SEIR-type model.

Our total model for all combined municipalities contains approximately 5.3 million people and simulates dynamics of spread through social interactions in layers such as households, schools and workplaces.

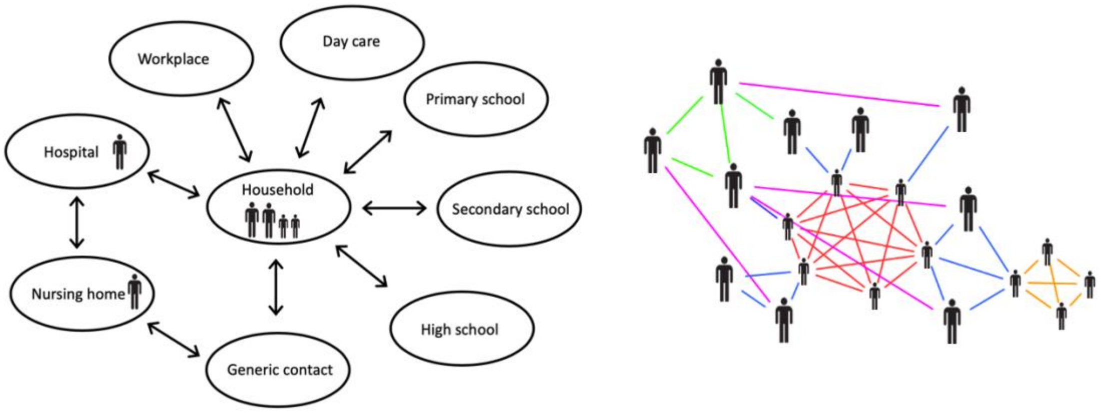

Supplementary Figure 1. Structure of network IBM. (Left) Possible domicile indicated by person figure. Named circles show available layers that a person can be member of. (Right) Illustrative example of resulting connection network between individuals caused by shared group membership in different layers: household (blue), primary school (red), day care (orange), work (green) and generic (pink).

Stochastic IBM network with SEIR epidemiological transmission dynamics

Structure of the municipality IBM network

We generate a high-fidelity IBM for a single municipality by creating a set of households corresponding to the population N_m of that municipality. Each household consists of one or several nodes (people), to each of which we assign a list of attributes:

1. Age
2. Domicile
3. Layer memberships / group membership within layers (see Supp. Figure 1)
4. Disease state (see Supp. Figure 2), and date of last change in disease state
5. Disease test state

The model has 9 layers. Layer a)-h) each consist of many groups, and each individual is only member of one of these groups. Further, an individual can only be present in a single of the layers b)-h). The layers are: a) Household, b) Day-care, c) Primary school, d) Secondary school, e) High school, f) Workplace, g) Nursing home, h) Hospital, and i) Generic contact network. A group is designed as a k-clique, i.e. where all members of a group are in contact. The assignments that form the IBM contact

network are constrained such that known high-resolution demographic data for each municipality is matched:

1) The household layer consists of separate households with a size and age distributions that follow demographic data for that municipality.
2) The number of schools, type, and their student populations are based on demographic data.
3) The number of day care facilities is based on demographic data.
4) The number of nursing homes and their population sizes are based on demographic data.
5) If a household has multiple children in e.g. day-care age, these children are assigned the same day-care. Similar for primary and secondary schools. For high-school age, the assignment to school unit is random.
6) In the work layer, the number of companies and their sizes are based on demographic data. Note that this layer is intended to represent spread between co-workers. For professions with large amounts of exposure to the general public, contacts with customers are represented through the generic contact network.
7) The generic contact layer is designed to capture heterogeneity in a person’s daily contact patterns. While the other layers consist of fixed cliques, in the random layer, each node connects to a random selection of other nodes for each simulated day. For each connection between an infected and a susceptible node, there is a fixed probability of the susceptible node becoming infected. The daily number of connections for each node is redrawn each day, according to a uniform distribution ranging from 1 to a maximum number of daily contacts C_D, which is a node attribute assigned at the initiation of the simulation.
8) We include two different modes: (A) For young (< 20) and elderly (80+) we assign a maximum number of daily contacts C_D following a normal distribution, whereas the remaining age groups are assigned contacts following a combination of a normal and a power law distribution: Contact number is drawn from (i) normal distribution, (ii) power law before being added together. The latter generates larger contact heterogeneity.

9) Using data from Statistics Norway[1], adults with different work and household municipalities are assigned work locations in the correct municipality. Supp. Table 2 lists the data tables used.
10) When a person is committed to a hospital, they are removed from their domicile (household or nursing home).
11) When an infected person manifests a symptom, is confirmed COVID-19 positive, or an asymptomatic person is confirmed COVID-19 positive, the model assumes they will self-quarantine from activities in all layers except their domicile.

All individuals participate in the generic contact network, which is designed as a random time-dependent scale-free network to capture heterogeneity in contact patterns. A new instance of this network (per municipality) is generated new every day. Except for the generic contact network, each grouping in each layer is represented as a k-clique, where every individual is connected to all others in the group, thus representing a well-mixed group. Supp. Figure 1 shows a schematic of the IBM layers inside which there are smaller groups (left panel) and a simplified example of a possible resulting contact network (right panel). The probability of infection depends on which layer the infected contact is located. The specific values used for the parameters in each layer are detailed in Supp. Table 1. Note that the infection probabilities apply between all nodes in a given layer, and as such represent a combined probability of contact and infection. While we may assume that some layers would see an actual close contact between all members of a given clique for each day (for instance, households), other layers, such as schools and workplaces, are not likely to actually see this form of all-to-all contact on any given day. This is reflected in a substantially lower combined contact and infection probability for these layers.

[1] Statistisk Sentralbyrå (SSB). https://www.ssb.no/statbank/

Population size scaling

We developed a schema for scaling up the population size of a municipality that preserves its demographic distributions. The scale parameter γ determines the new size of a municipality: γ =1 for the unscaled population, γ = 0.5 results in a municipality with half of the population, and γ =2 gives a municipality with twice the original population. As the statistical data, age data and household-, nursing home- and workplace-compositions obtained from Statistics Norway take the form of percentages, the distributions are independent of the size of the network and can be used as provided, simply by multiplying by γ the number of desired individuals/households/workplaces (provided by Statistics Norway and passed directly as inputs to the generating code when $\gamma = 1$). On the other hand, schools and daycares are explicitly and individually defined, and their numbers are too few to be mapped to fine-grained size distribution. Therefore, in order to scale these systems to match the scaling of the whole municipality, we instead opt for an approach where we randomly select and duplicate (or erase, for $\gamma < 1$) schools and daycares in the original municipality data until we reach the number specified by the scale factor.

Variation of household-size distribution

In order to evaluate the impact of the household size distribution on spreading potential, we needed to establish a systematic way to modify the distribution, preferably defined by a single scalable parameter α. We decided on either splitting or merging existing a fraction of households in the original distribution, yielding smaller or larger households respectively. In the first case, we identify households eligible for splitting, using the criteria that it must contain at least two adults. This is necessary in order to ensure that we do not create households containing only children. We then define α as the fraction of eligible households to split. The split itself is conducted by assigning the two first adults in the selected household to their own household, with each of the remaining individuals assigned to either household by coin flip (Bernoulli, 0.5).

We represent the merging of households by using negative values of α: As merging households with at least one adult each will naturally lead to a new household with at least one adult, all households are eligible for merging. We therefore randomly assign the households into pairs, with a chance |α| that determines the fractions of pairs which are combined to form a single household consisting of the members of the two original households, reducing the total number of households in the system by one for each merged pair. The households in the remaining pairs stay unchanged.
As an example, consider a fictitious small municipality containing 2000 individuals, labeled *i1, i2*, *i3*, … , distributed across 1000 households *H1, H2, H3, …* such that *H1* contains individuals *i1, i2* (using the notation *H1 = [i1, i2], H2 = [i3]* (a lone individual), *H3 = [i4, i5, i6, i7]* , and *H4 = [i8, i9].* We then pair household H1 with household H2 and household H3 with household H4 and label this list *X*, so that *X1 = [H1, H2] = [i1, i2, i3], X2 = [H3, H4] = [i4, i5, i6, i7, i8, i9], ….* Our next step is to initiate an empty list of new households, which we label *N*. We take *X1,* and with probability |α| add *N1 = [i1, i2, i3],* otherwise we add *N1 = H1 = [i1, i2]* and *N2 = H2 = [i3]*. We repeat this process for *X2,* adding *H3* and *H4* to *N* either separately (with probability 1-|α|) or as one combined household *[i4, i5, i6, i7, i8, i9]* (probability |α|)*,* and similarly for each pair in X. If the number of households is odd, one household is copied directly into *N* without possibility of pairing. The remaining households are assigned to pairs as described.

Note that *N* will have a lower total number of households than *H*, but the same total number of individuals*.* For instance, setting α = 0.1 in a network starting with 1000 households, the average result is *N* consisting of 950 households (900 households in 450 pairs remain unchanged, with the remaining 100 households merged pairwise into 50 new ones). For α = 0.5 in a network starting with 1000 households, we would get (on average) 750 households, with 500 households unchanged and the other 500 being combined pairwise in order to form 250 households.

SEIR-type epidemiological dynamics

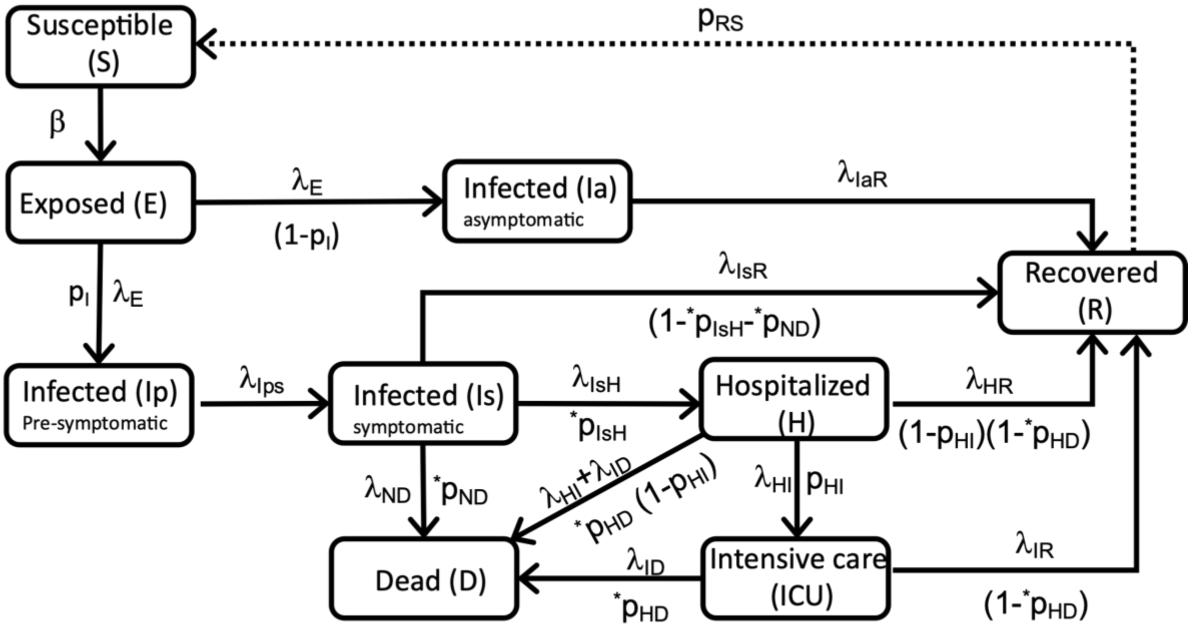

Supplementary Figure 2. Block diagram showing schematic of the SEIR-type epidemiological state model for each individual in the IBM. Solid lines show transitions currently active in the model. The dotted line indicates current uncertainty about long-term COVID-19 immunity. Starred parameters indicate age dependent values.

Each individual in the IBM model is either healthy, in various states of infection, or recovered from the disease: Susceptible (S); Exposed (E); Infected, asymptomatic (Ia); Infected pre-symptomatic (Ip); Infected, symptomatic (Is); Hospitalized (H); Intensive care (ICU); Recovered (R); or Dead (D). The different states and their possible transitions are captured by the state-transition schematic of Supp. Figure 2.

Pre-symptomatic and asymptomatic individuals are infectious and active in all layers they are associated with (for most, this will be the generic contact layer, a household, and either a workplace or educational/daycare institution). Symptomatic individuals are assumed to be infectious in the household and nursing home layers only. Hospital and ICU protective measures are assumed to be sufficient to prevent spread from patients to staff.

The direct transition from Is to D captures the disease trajectory for some people in nursing homes. The transition from S to E is governed by disease transmission probabilities in the IBM contact network. After an individual is infected from a neighbor in the contact network, the SEIR-type dynamics of that individual’s disease state (onward from E) are governed by stochastic processes with appropriate waiting times according to empirical data for COVID-19. We continually update our parameter estimations given the daily update in disease state numbers for Norway by region.

At each time point, an individual will store four points of information about their progression through the SEIR states: current state, date of last change of state, next state, and date of next change of state. Each day, the model updates the state as needed. Upon entering a new state, the model selects the following state according to the probabilities and waiting times specified in Supp. Figure 2 and Supp. Table 1. Once the next state is determined, we generate the duration of the state according to a Poisson-distributed random variable (with an appropriate λ) plus 1 (in order to avoid two state changes in one day) and set the date of next change of state accordingly.

Note that our SEIR-model uses age-stratified transition rates, where the age of an individual decides which of the age groups that individual is part of Supp. Table 1 shows the rates used for the different SEIR transitions of Supp. Figure 2.

COVID-19 testing of (sub-) population

The testing itself is represented by drawing individuals from the symptom-free population (susceptible, latent, asymptomatic, pre-symptomatic, or recovered), and returning a positive test if they are asymptomatic or pre-symptomatic. Pooled tests are represented similarly, but with multiple people (forming a pool) simultaneously tested, and all tests marked as positive if any of the people in the test pool are in the asymptomatic or pre-symptomatic states. Our IBM implementation

allows for random selection of individuals for testing, or testing based on a pre-conceived schema for node attributes.

Base-line fitting of IBM to empirical data

The parameters in the stochastic IBM are treated as fixed, and they are either set from literature or estimated directly from publicly available empirical data, as indicated in Supp. Table 1. We determined model parameters by fitting the predicted hospitalization rate of our Oslo model to hospitalizations for Oslo in the period from March 1st to April 20th. We assume a sudden transition between two regimes, with infections following original “unrestricted” probabilities until March 13th. The “unrestricted” regime is initialized with 20 cases at an unspecified date and run until the system reaches N_{lock}= 400 active cases. This point is marked as March 13th and serves as a reference point for the remainder of the simulation. The motivation for fixing dates at this point (rather than the initial point of 20 cases) is two-fold: First, it is important that the model follows a progression of non-pharmaceutical interventions (NPIs) that actually corresponds to those implemented by Norwegian authorities. Therefore, we decided to use the earliest NPIs introduced (March 13th) as an anchoring point, with the dates of subsequent NPIs defined in relation to the first. Second, due to the heterogeneity in the infectious potential of individuals (with a small fraction of individuals being substantially more infectious than others due to their particular contact network), the model exhibits highly stochastic behavior when the number of infections is low, as the number of high-infectivity individuals infected is more variable. As small fluctuations in the early evolution of the epidemic can have dramatic effects on long-term forecasts, anchoring the network around a reasonably large number of cases allows the model to make more consistent predictions. This approach also solves the issue of highly stochastic behavior at low infection levels, as 400 active cases is sufficient to reduce this notably.

After these steps, all schools and day care facilities close, as do 50% of workplaces. Random contacts are reduced by 83%. Infection probabilities in nursing homes and within-households remain the same in both regimes. Model simulations start in March and continue until August 30th, 2020.

Model fitting to the data was then done by adjusting infection probabilities in order to satisfy the following criteria: (1) matching slopes (both increasing and decreasing) and (2) the location (date) and duration of the peak of the hospitalization data (Fig 2A, red curve), (3) actual hospitalizations fitting between confidence intervals for the duration of the simulation. Additionally, we checked that (4) the fraction of computed household infections was within the 35-45% band seen in the officially reported weekly values, as well as approximate accordance with the relative share of infections caused by each of the other layers as identified by Norwegian contract tracing efforts and compiled by the Norwegian Institute of Public Health (FHI): 3% in school settings (4-5% in model) 18% in workplace/university settings (4-10% in the model), 40% others (45-50% in the model). Note that the model definition of workplace contact involves repeated interaction between individuals working in the same location, and is therefore more restrictive than the FHI definition which includes customer-to-worker infections (which are counted as random layer infections in the model). Fitting was done by adjusting infection probabilities alone until a satisfactory fit (by visual judgment) was reached. While the epidemic curve is also affected by state durations, these were kept fixed based on reliable direct clinical data which was readily available (as substantial deviations from these would in any case be hard to justify).

Since the lockdown in Norway occurred in the early stages of the epidemic, the fitting of the IBM results to data depends very much on the number of symptomatic infected on the day of lock down (March 13, 2020). Thus, for a given parameter set and municipality, we have used a large number of simulations to determine the optimal number of infected (N_{lock}) on this day for the model predictions to fit to time series data of number of hospitalized persons. Simulations are

subsequently conducted by initiating the network with a small number of infections and running the code until N_{lock} is reached. As described above, the date is set to be March 13 at this point. We anticipate that several of the IBM parameters can be estimated from other data sources when they become available, e.g. large-scale systematic testing regimes for COVID-19.

Description of the TPHT process

Selection of households

The central aspect of TPHT is the prioritizing households for testing according to size. As an example, consider a municipality with the following distribution of households:

- 6 persons and over: 3,000 households
- 5 persons: 7,000 households
- 4 persons: 30,000 households
- 3 persons: 20,000 households
- 2 persons: 50,000 households
- 1 person: 90,000 households.
- Total: 200,000 households, population of above 420,000 (depending on the exact distribution of households of size 5 and more)

In this case, a TPHT test fraction of 2.5% corresponds to 5,000 households, meaning we select all households 6 and over (3,000), and a random choice of 2,000 of the 5-person households. A TPHT test fraction of 5% would yield 10,000 households, meaning all households 5 and above. A TPHT test fraction of 10% gives 20,000 households, meaning all 10,000 households five and above, as well as 10,000 randomly chosen 4-person households. For a given fraction and test intervals, the selected households are distributed evenly across the interval. For instance, using a test fraction of 3.5% on the municipality above with a test interval of 7 days, we would have to test 7,000 households a

week; with 1,000 periodically tested every Monday, another 1,000 tested every Tuesday, and so on for the duration of the TPHT test regime.

Pooled household testing and quarantine

Pooled testing is a method by which samples from multiple individuals (a pool) are combined and jointly analyzed, returning a positive if any of the individuals constituting the pool are infected, without giving further information as to *which* of the individuals in the pool are infected (*Sunjaya & Sunjaya*, 2020).

Due to the household constituting a high-risk environment for transmission that is not mitigated by many of the interventions introduced to curb epidemic spread, such as work from home, school shutdowns, closing of public venues and so on (weekly reports from the Norwegian Institute of public health show a relatively constant 40-45% of infections happening in a household setting throughout the pandemic), TPHT proposes treating each household as a common unit with regards to testing of isolation. In short, if one household member is confirmed to be sick, all household members should be treated as if they were, preventing the infection from being passed on through society at large through undiagnosed family members.

Estimation of R for a choice of simulation parameters

Due to the individual-based nature of our model, each infection can be traced to the individual causing it. This also makes it possible to keep count of the number of secondary infections caused by each infected individual, which should make computing R fairly straightforward. However, there are some caveats. We want to determine R for a given point in time, but a closer consideration of the definition of the reproduction number reminds us that it is the number of secondary cases generated by an original case, and as

such the strict definition of R applies to an infected population rather than a point in time. We can, however, define a daily reproduction number by taking the average number of secondary cases caused by each individual infected on a given day. This number cannot be computed in real time, as any individual that does not recover on the specified day may very well cause additional infections before it recovers. Therefore, we opt to determine R through a “two-pass” process. We begin by simulating the epidemic over a given time interval in a first pass, assigning to each infected individual (except those which have not completed the course of the disease by the end of the simulation interval) an individual reproductive number as well as noting their infection and recovery dates. In the second pass, then re-iterate through every day, determining an average daily reproductive number by taking the arithmetic mean of the individual reproductive numbers for everyone not in the S or R states on that day.

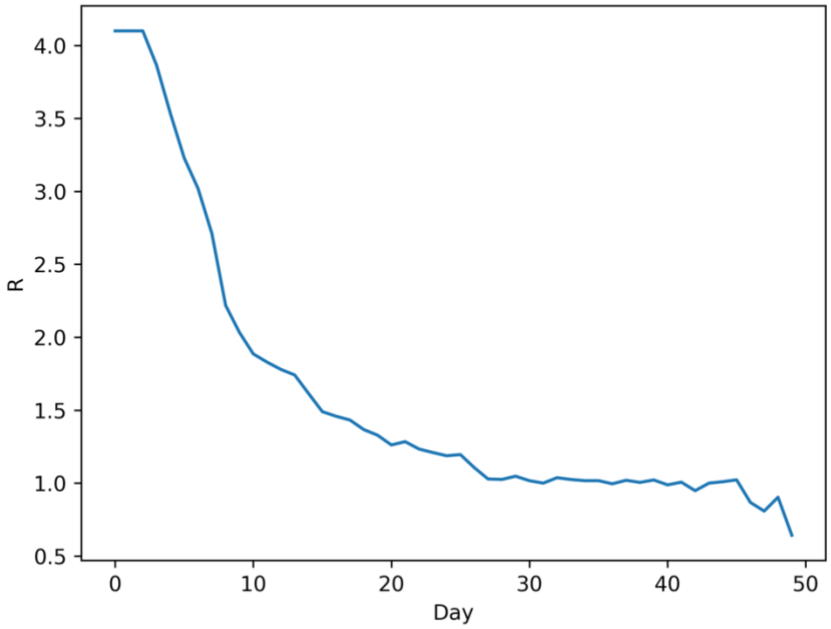

Supplementary Figure 3: Daily estimated R for a test regime consisting of 10 days with early March parameters, with an abrupt transition to late May parameters at t=10 days and on.

We make three key observations: first, there is some stochasticity in the determined value of *R* between consecutive days; second, the transition from one regime to the next exhibits an intermediary phase of a few days until *R* begins to stabilize around a new average. We propose two primary explanations for this. Firstly, as we compute R by taking the average number of infections caused by all individuals at a chosen time, individuals that were already infected before measures were introduced will have been more infectious during that period, driving up the average. Second, different layers are unevenly affected by measures. Consider a transition from free spread to a regime where only workplace spread is possible, but school, random, nursing home and even household spread is entirely prevented. As restrictions enter into force, a few workplaces will have active cases. These will cause further infections for some time, either gradually petering off or saturating each environment, in which case R would abruptly drop to zero.
Lastly, we see a more abrupt drop-off in the estimated reproductive number as we reach the last few days of the simulation period. This is because many of the last nodes to be infected do not have time to recover, and with the requirement that a node must recover in order to have its reproductive numbe included in the average, there is a bias towards individuals with shorter illnesses, which pulls down the average R.

Meanwhile, estimating R over longer periods of time leads to substantial discrepancies: if R > 1, there is a build-up of immunity, leading to a gradual reduction in R; if R < 1, infection levels will gradually drop off, leading to situations with very few infected and correspondingly high noise in the relative number of new daily infections. Because of this, for purposes of determining R, our process is as follows:

- First, initialize the model with 20 infected individuals
- Unfettered growth until 100 symptomatic cases are reached (this is enough to remove most of the noise, while not enough to build up substantial immunity in the population). The

purpose of this approach (rather than just beginning with a 100 infected individuals on the first day) is described in the next section.

- Once 100 (simultaneous) symptomatic cases are reached, transition into the regime for which we wish to determine R, and run this for 40 days. Due to the high infectivity up to this point, there is also a substantial number of pre-symptomatic cases already infected at this point.
- Take the daily average R from 17 to 22 days after the transition mentioned above (equivalent to the 27- day interval in Supp. Figure 3 above). All figures are based on 40 replicate runs for each configuration.

Simulation of outbreaks

When conducting the simulation of an outbreak consisting of N_{out}=1,000 symptomatic infected, the simple approach is to randomly select N_{out} nodes in the network and abruptly change their state from susceptible to exposed. However, this will cause artificial oscillations in the data since all of the exposed individuals start out in synchrony because they were infected at the same time. It takes several infection generations for these oscillations to subside. Instead, we dynamically generate N_{out} by starting from a small seed of 20 randomly (simultaneously) selected and exposed individuals. Subsequently, the IBM is simulated until we reach N_{out} symptomatic individuals. This approach for achieving N_{out} does not cause infections to be focused on larger households, instead spreading them randomly and asynchronously in the network due to the large fraction of infections taking place in the random contact layer. The state with N_{out} symptomatic individuals is used as the starting point (day 0) of the outbreak simulation, from which interventions may be implemented. We go through this initiation process independently for each separate simulation, thus ensuring stochastic variation in the day 0 state.

Software availability

The IBM software used for performing the simulations is available on two general access platforms:

1. Active development on GitHub: https://github.com/andrevo/covid19-ntnu
2. Code snapshot used to generate the results of this publication are available at Figshare following DOI: 10.6084/m9.figshare.14551020

Supplementary Figure 4. Effect of TPHT for alternative COVID-19 disease parameters

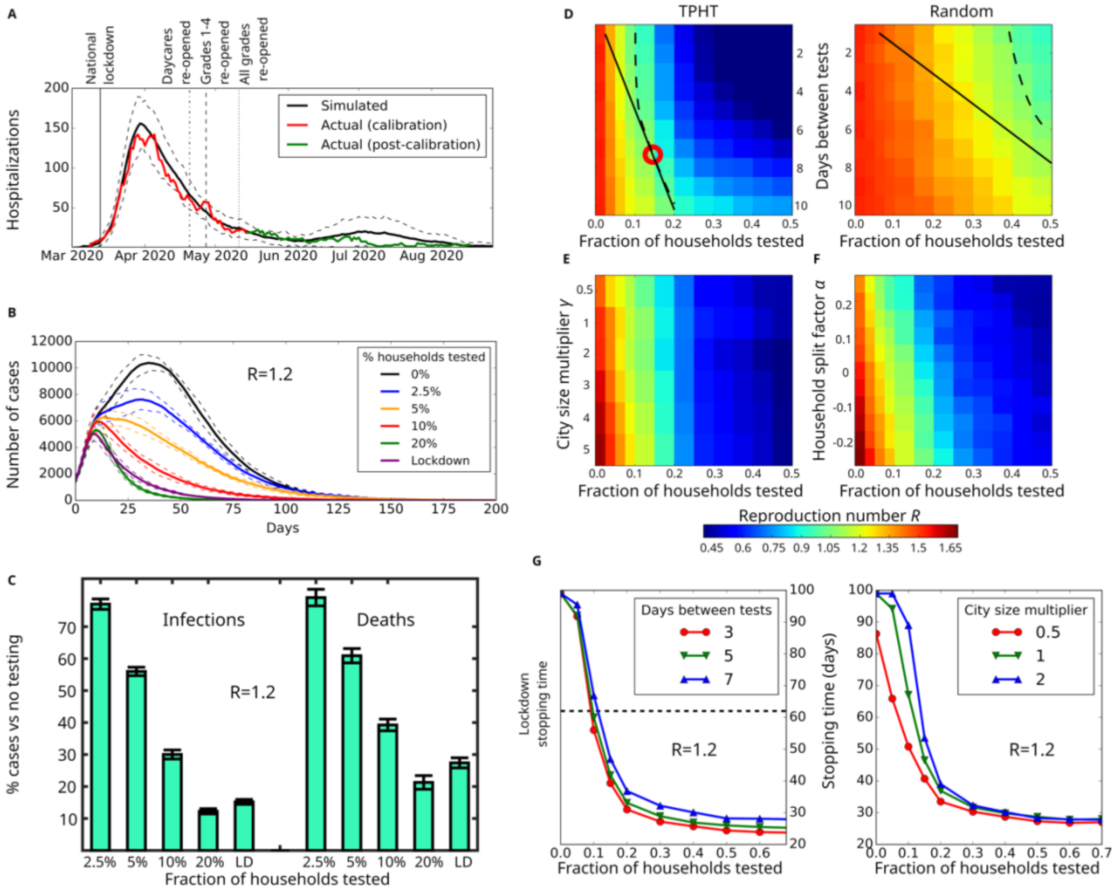

Caption: Simulation results and effectiveness of targeted pooled household testing (TPHT) on COVID-19 using alternative COVID-19 disease parameters (see Supp. Table 1 for values in red). (A) Fitting the model to Oslo hospitalization data. We plot the mean predicted number of hospitalizations (black) and confidence interval (2σ, dashed). Actual Oslo hospitalizations (red) were used as calibration (until May 15th). Hospitalization data May 15th- August 30th (green) were not used to determine model parameters. (B) Effect of TPHT in response to a sudden rise in cases (reaching 1,000 symptomatic individuals), assuming general infectivity parameters similar to those of Oslo in late May 2020 but with 75% increased infectivity of random contacts giving R=1.2. (C) Predicted number of deaths and infections for different TPHT testing fractions corresponding to panel (B), relative to no testing. Panels (D)-(F) use same parameters as panel (B), except with a 113% increased infectivity of random contacts giving R=1.4. (D) Effect of test frequency and fraction on R,

for TPHT (left) and random pooled household testing (right). Dashed and solid lines indicate isoclines for R=1 and constant test density, respectively. Optimal point is marked with red circle. (E) Response of weekly TPHT to varying city size. We scale the population of the baseline Oslo model (γ=1) to generate larger (γ>1) or smaller networks with household, school, daycare, workplace and nursing home size-distributions unchanged. (F) Response of weekly TPHT to changes in distribution of household size, relative to the baseline Oslo model (α=0). For α>0, a portion of households are each split into a new pair, yielding a smaller average household size than the baseline. For α<0, a portion of the households are pairwise merged, yielding a larger average household size than the baseline. Household, school, daycare, workplace and nursing home size-distributions are kept unchanged. (G) COVID-19 stopping time (number of days until symptomatic cases are reduced by 75%) in response to changing days between tests (left) and fraction of weekly TPHT tests (right). Stopping times longer than 100 days are truncated.

Supplementary Figure 5. Household-size histograms.

When varying the household-size composition in the model, we used the approach detailed above in this document. Here, we show three of the resulting household-size histograms for α=-0.3, 0, 0.3. Note that the bars sum up to 700,000 for each of the α–values.

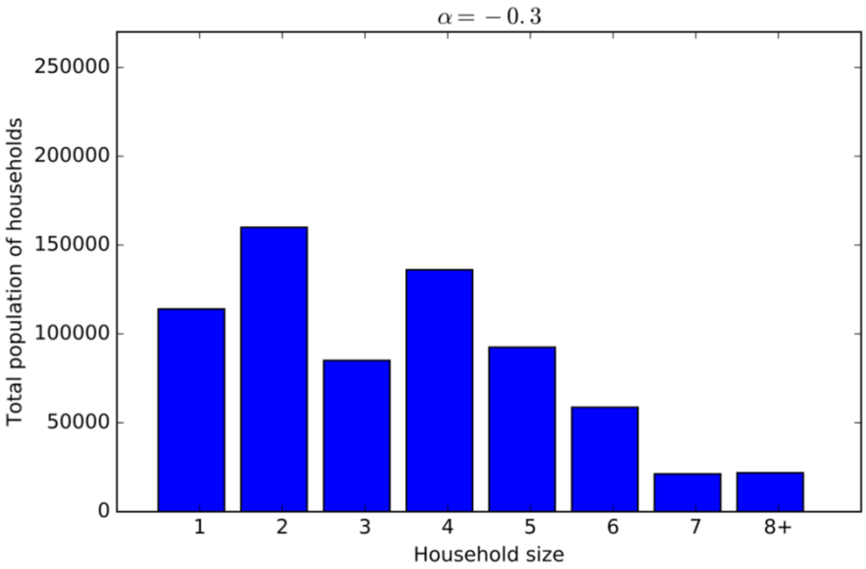

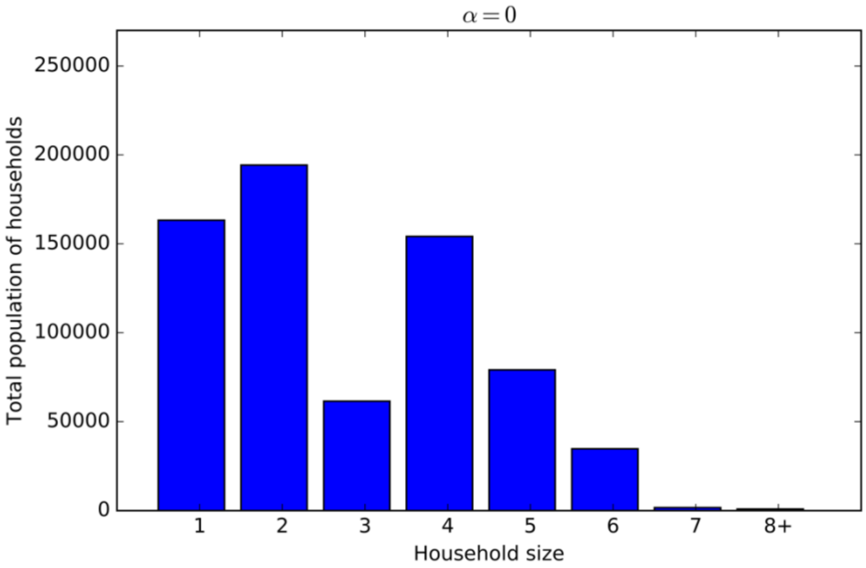

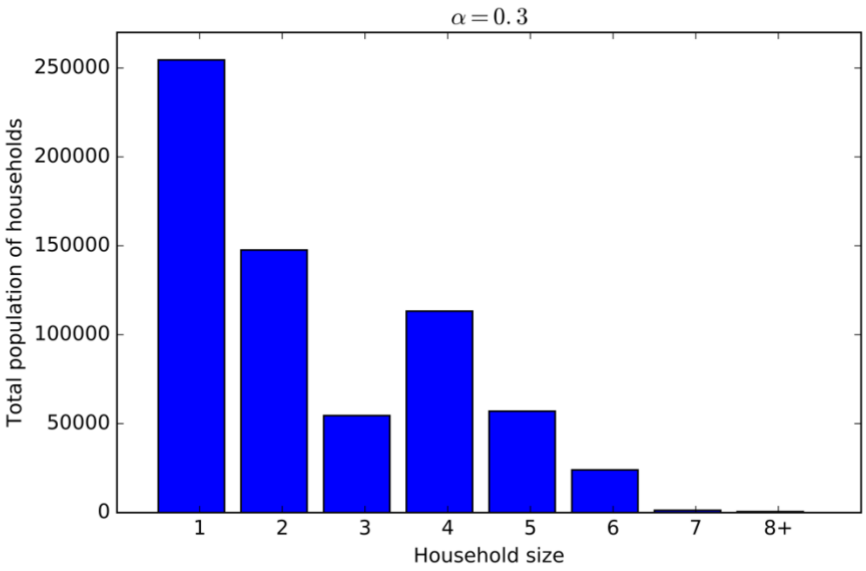
$\alpha = 0.3$
Total population of households
250000
200000
150000
100000
50000
0
1
2
3
4
5
6
7
8+
Household size

Supplementary Figure 6. Effect of compliance on the efficacy of TPHT.

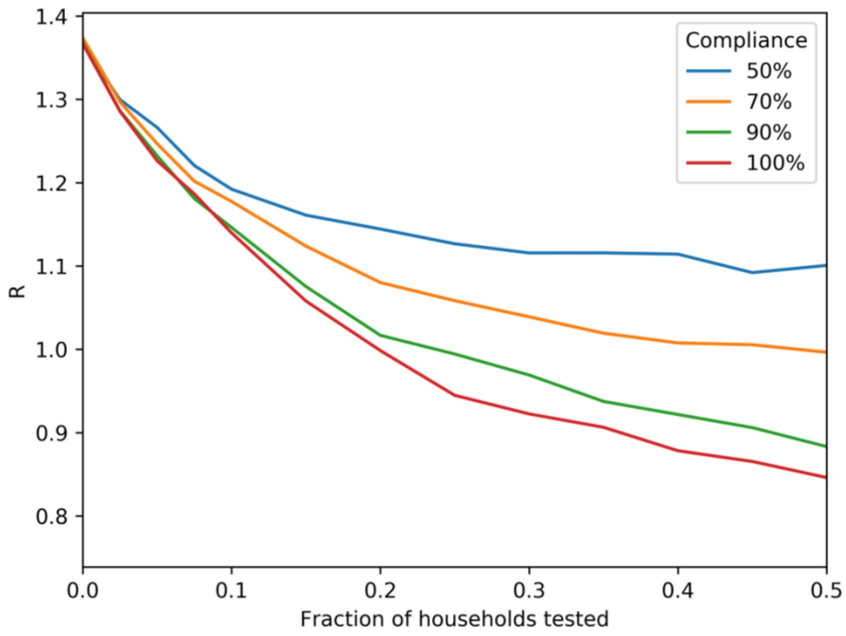

R as a function of test fraction with 50%, 70%, 90% or 100% of households willing to participate in TPHT. The testing frequency is set to once every 7 days for each of the four curves. The x-axis corresponds to the number of households actually tested (and therefore actual burden on testing capacity). For instance, the number of tests actually administered for a test fraction of 0.1 is the same for 50% and 100%; however, 50% compliance means that half of the 20% of largest households are tested. In contrast, 100% compliance means that all of the 10% largest households are tested weekly.

Supplementary Table 1. Parameters used in simulations

Model parameters	Symbol	Value	Function	Source
**SEIR-epidemics**				
Probability of infection	b	----	Network effect	
Days incubation time	λ_E	4 (1)	Fixed	
Days spent pre-symptomatic	λ_{Ips}	2 (5)	Poisson	Norwegian Institute of Public Health (FHI)
Days symptomatic before recovery	λ_{IsR}	5	Poisson	Norwegian Institute of Public Health (FHI)
Days symptomatic before hospitalization	λ_{IsH}	6	Poisson	Data from Health Region South East, Norway
Days symptomatic in nursing home before death	λ_{ND}	14 (10)	Poisson	Data from Health Region South East, Norway. Presumed equal to days from symptom onset to death for hospitalized cases.
Days in hospital before recovery (no ICU)	λ_{HR}	4 (8)	Poisson	Data from Health Region South East, Norway
Days in hospital before ICU	λ_{HI}	4	Poisson	Data from Health Region South East, Norway
Days in ICU before recovery	λ_{IR}	8 (12)	Poisson	Data from Health Region South East, Norway
Days in ICU before death	λ_{ID}	8 (12)	Poisson	Data from Health Region South East, Norway
Days asymptomatic before recovery	λ_{IaR}	8	Poisson	Norwegian Institute of Public Health (FHI)
% exposed developing symptoms	P_I	50	Bernoulli	
% symptomatic dying outside of hospital:	P_{ND}		Bernoulli	
nursing home residents 70-79 years		26		Adjusted to Norwegian hosp. death rates, Verity et al
nursing home residents 80-89 years		42		Adjusted to Norwegian hosp. death rates, Verity et al
All others		0		
% hospitalized dying:	P_{HD}		Bernoulli	Verity et al, Lancet, 2020
0-9 years		1.61 e-3		
10-19 years		6.95 e-3		
20-29 years		3.09 e-2		
30-39 years		8.44 e-2		
40-49 years		0.161		
50-59 years		0.595		
60-69 years		1.93		
70-79 years		4.28		
80+ years		7.8		
% symptomatic being hospitalized:	P_{IsH}		Bernoulli	Verity et al, Lancet, 2020
0-9 years		0		

10-19 years		0.048		
20-29 years		1.04		
30-39 years		3.43		
40-49 years		4.25		
50-59 years		8.16		
60-69 years		11.8		
70-79 years		16.6		
80+ years		18.4		
% hospitalized needing ICU	P_{HI}	30	Bernoulli	Fitted to Norwegian Institute of Public Health, ICU numbers
% not developing immunity	P_{RS}	0	Bernoulli	
**Individual-based network model**				
Infectiousness in Day Care		0.015%	Bernoulli	estimated IBM fit to Norwegian clinical data
Infectiousness in Primary School		0.010% (0.002%)	Bernoulli	estimated IBM fit to Norwegian clinical data
Infectiousness Secondary School		0.010% (0.015%)	Bernoulli	estimated IBM fit to Norwegian clinical data
Infectiousness High School		0.010% (0.015)	Bernoulli	estimated IBM fit to Norwegian clinical data
Infectiousness Household		5.0% (15.0%)	Bernoulli	estimated IBM fit to Norwegian clinical data
Infectiousness Work		0.015%	Bernoulli	estimated IBM fit to Norwegian clinical data
Infectiousness Nursing Home		15.0% (20.0%)	Bernoulli	estimated IBM fit to Norwegian clinical data
Infectiousness Generic Contact		2.00% (1.15%)	Bernoulli	estimated IBM fit to Norwegian clinical data
Pre-symptomatic case infectiousness multiplier		3 (1)		Adjusted to CDC estimate (planning scenario 5) for proportion of infections before symptom onset
Asymptomatic case infectiousness multiplier		0.5 (1)		Norwegian Institute of Public Health
Relative infectivity and susceptibility of children under 13		0.5 (1)		Estimated IBM fit to Norwegian clinical data
Maximum daily contacts, mean of normal distribution		10		estimated IBM fit to Norwegian clinical data
Maximum daily contacts, variance of normal distribution		3		estimated IBM fit to Norwegian clinical data
Maximum daily contacts, exponent of power law		-0.5		estimated IBM fit to Norwegian clinical data

Parameter values in black are used in computer simulations for Fig. 2 in the main article. To generate Supp. Figure 4, parameter values in red were used instead when present in the table. The infection probabilities given under the section “Individual-based network model” correspond to daily secondary attack rates in a clique with one symptomatic individual (except for the generic layer,

which is not clique-based). For example, in a household with one symptomatic infected individual, each other individual has a 5% chance of being infected per day. The infectivity of pre-symptomatic and asymptomatic individuals for a given type of interaction are adjusted by a factor 3 and 0.5, respectively. Children are half as likely to contract the disease from a given interaction. In cliques with multiple infected individuals, the probability increases multiplicatively. For example, consider a household with two symptomatic, one asymptomatic and one pre-symptomatic case (all adult). Here, the daily infection probability for remaining household members becomes:

$P = 1-(1-0.05)*(1-0.05)*(1-0.025)*(1-0.15) = 0.25$

which corresponds to 25%.

The specified infectiousness of generic contacts is the base probability of transmission from a single random contact.

Supplementary Table 2. Demographic data used to generate layered network.

In generating the layered network with high-resolution demographic data for each municipality in Norway, we used the data tables from Statistics Norway and from the Norwegian National School Registry (API: https://data-nsr.udir.no/, contains complete information for all schools).

The following Statistic Norway data tables are all available by accession number from https://www.ssb.no/en

Accession number	Data table text description
3321	Employed persons (aged 15-74) per 4th quarter, by municipality of work, municipality of residence, contents and year
4469	Residents in dwellings for nursing and care purposes, by age (M) 2002 - 2019
6070	Private households, by type of household (M) 2005 - 2019
6079	Private households and persons in private households, by size of household (per cent) (M) (UD) 2005 - 2019
6206	Children 0-17 years, by number of siblings and the child's age 2001 - 2019
6445	Employed persons, by place of residence, sex and age (per cent). 4th quarter (M) 2005 - 2019
8947	Pupils, apprentices, students and participants in upper secondary education, by sex, age and type of school/institution 2006 - 2019
9169	Children in kindergartens, by age, hours of attendance per week and ownership (M) 1999 - 2019
9220	Kindergartens, by ownership (M) 1987 - 2019
9929	Nursing and care institutions and beds, by ownership (C) 2009 - 2018
10308	Establishments, by the enterprises sector and number of employees (M) 2012 - 2020
11933	Care institutions - rooms, by region, contents and year
12562	Selected key figures kindergartens, by region, contents and year

Supplementary Table 3. List of 15 demographic variables studied in epidemic data from Santé publique

**Variable name**	**Spearman**
% households >4 people	0.54
Population share aged 0-4	0.54
Department longitude	0.53
Population share aged 60+	-0.53
Population share aged 20-39	0.51
Department latitude	0.48
% households >3 people	0.46
Household mean people number	0.40
Department population density / sq. km	0.38
Populationshare aged 5-19	0.36
Department area / sq. km	-0.32
Department population	0.28
% households >2 people	0.19
% households >1 people	0.13
Population share aged 40-60	-0.02

Source for data:

1. Santé publique: Download Sept. 21, 2020 https://www.data.gouv.fr/fr/datasets/donnees-hospitalieres-relatives-a-lepidemie-de-covid-19/
2. French National Institute of Statistics and Economic Studies - Households according to size in 2017. http://www.alisse2.insee.fr/fr/statistiques/2012714
